# Ultrasound Molecular Imaging of Secreted Frizzled Related Protein-2 Expression in Murine Angiosarcoma

**DOI:** 10.1371/journal.pone.0086642

**Published:** 2014-01-29

**Authors:** James K. Tsuruta, Nancy Klauber-DeMore, Jason Streeter, Jennifer Samples, Cam Patterson, Russell J. Mumper, David Ketelsen, Paul Dayton

**Affiliations:** 1 Department of Pediatrics, University of North Carolina at Chapel Hill, Chapel Hill, North Carolina, United States of America; 2 Department of Surgery, University of North Carolina at Chapel Hill, Chapel Hill, North Carolina, United States of America; 3 UNC McAllister Heart Institute, University of North Carolina at Chapel Hill, Chapel Hill, North Carolina, United States of America; 4 Joint Department of Biomedical Engineering, University of North Carolina at Chapel Hill, Chapel Hill, North Carolina, United States of America; 5 North Carolina State University, Raleigh, North Carolina, United States of America; 6 Division of Molecular Pharmaceutics, UNC Eshelman School of Pharmacy, Chapel Hill, North Carolina, United States of America; Northwestern University Feinberg School of Medicine, United States of America

## Abstract

Angiosarcoma is a biologically aggressive vascular malignancy with a high metastatic potential. In the era of targeted medicine, knowledge of specific molecular tumor characteristics has become more important. Molecular imaging using targeted ultrasound contrast agents can monitor tumor progression non-invasively. Secreted frizzled related protein 2 (SFRP2) is a tumor endothelial marker expressed in angiosarcoma. We hypothesize that SFRP2-directed imaging could be a novel approach to imaging the tumor vasculature. To develop an SFRP2 contrast agent, SFRP2 polyclonal antibody was biotinylated and incubated with streptavidin-coated microbubbles. SVR angiosarcoma cells were injected into nude mice, and when tumors were established the mice were injected intravenously with the SFRP2 -targeted contrast agent, or a control streptavidin-coated contrast agent. SFRP2 -targeted contrast agent detected tumor vasculature with significantly more signal intensity than control contrast agent: the normalized fold-change was 1.6±0.27 (n = 13, p = 0.0032). The kidney was largely devoid of echogenicity with no significant difference between the control contrast agent and the SFRP2-targeted contrast agent demonstrating that the SFRP2-targeted contrast agent was specific to tumor vessels. Plotting average pixel intensity obtained from SFRP2-targeted contrast agent against tumor volume showed that the average pixel intensity increased as tumor volume increased. In conclusion, molecularly-targeted imaging of SFRP2 visualizes angiosarcoma vessels, but not normal vessels, and intensity increases with tumor size. Molecular imaging of SFRP2 expression may provide a rapid, non-invasive method to monitor tumor regression during therapy for angiosarcoma and other SFRP2 expressing cancers, and contribute to our understanding of the biology of SFRP2 during tumor development and progression.

## Introduction

Angiosarcoma is a biologically aggressive vascular malignancy with a high metastatic potential and subsequent mortality [1]. It originates from endothelial cells of small blood vessels and may affect a variety of organs, including the retroperitoneum, skeletal muscle, subcutis, liver, heart and breast. The outcome of angiosarcoma is poor for those patients in whom aggressive surgery cannot be considered, and therefore there is a desperate need for novel therapies to improve survival in patients with this highly lethal disease. A better understanding of the biology of angiosarcoma is needed to identify new molecular targets.

The DeMore laboratory has recently discovered a novel angiogenesis factor involved in angiosarcoma growth. While conducting genomic profiling of breast tumor vascular cells obtained by laser capture microdissection, secreted frizzled related protein 2 (SFRP2) was identified as a gene with 6-fold increased expression in tumor endothelium as compared to normal vessels [2]. SFRP2 is a 33 kDa secreted protein involved in the Wnt signaling pathway, an important pathway in tumor biology [3]. Since angiosarcomas have been reported to represent the signaling abnormalities of pathogenic angiogenesis [4], we speculated that SFRP2 would also be expressed in human angiosarcomas, which we confirmed by immunohistochemistry [5]. SFRP2 acts as a novel stimulator of angiogenesis *in vivo* and *in vitro* by stimulating endothelial cell migration, protecting against apoptosis, and is required for and stimulates angiosarcoma tube formation [5]. We recently reported the generation of a murine monoclonal antibody to SFRP2 that inhibits angiosarcoma allograft and breast cancer xenograft growth in vivo [6]. Thus, SFRP2 is a novel therapeutic target for angiosarcoma and other tumors.

Although SFRP2 is a secreted protein, it has been demonstrated to incorporate into the extracellular matrix [7] and localizes to tumor endothelium [2]. Thus we hypothesized that SFRP2-directed imaging could be an approach to imaging the tumor vasculature. Currently, tumor response following drug treatment is based on measurement of anatomical size changes [8]. However, the standard response measurement does not provide insight into changes of molecular characteristics. In the era of targeted medicine, knowledge of specific molecular tumor characteristics has become more important. Molecular imaging using targeted ultrasound contrast agent can monitor tumor progression non-invasively [9]. The principle behind ultrasonic molecular imaging is the selective adherence of microbubble contrast agents to biomarkers expressed on the endothelium [10]. Once the contrast agents accumulate at the target site, they enhance the pathologic tissue via increased acoustic backscatter, thus visualizing the presence of biomarkers associated with disease [11]. This approach evaluates biological changes at the molecular level before measurable anatomic changes occur. In this study we report the development of a new molecular imaging reagent to non-invasively monitor the progression of angiosarcoma by targeting SFRP2 in the tumor vasculature. In addition to a potential clinical imaging application, this technology allows us to further elucidate the biology of SFRP2 in tumor progression.

## Materials and Methods

### Cell culture

Murine SVR angiosarcoma cells were obtained from American Type Culture Collection (ATCC®, Manassas, VA) and cultured in low-glucose DMEM with 10% fetal bovine serum (FBS) (Sigma-Aldrich, St. Louis, MO). ATCC provides authenticated cell line identity, and in addition, SVR angiosarcoma cells were tested negative by Research Analytic Diagnostic Laboratory (Columbia, MO) for PCR evaluation for: Ectromelia, EDIM, LCMV, LDEV, MHV, MNV, MPV, MVM, *Mycoplasma* sp., Polyoma, PVM, REO3, Sendai, TMEV GDVII. Cells culture was carried out at 37°C in a humidified 5% CO_2_-95% room air atmosphere.

### Ethics Statement

This study was carried out in strict accordance with the recommendations in the Guide for the Care and Use of Laboratory Animals of the National Institutes of Health. The protocol was approved by the Institutional Animal Care and Use Committee (IACUC) for the University of North Carolina at Chapel Hill approved all animal procedures (IACUC ID No. 12-125.0). Animals were anesthetized with isoflurane prior to tumor imaging. All efforts were made to minimize suffering.

### Size-sorted ultrasound contrast agent

A lipid solution containing an 18∶1∶1 molar ratio of DSPC, PEG2000-PE, PEG2000-PE-Biotin was sonicated to produce lipid encapsulated perfluorobutane micro bubbles as described previously [12]. Differential centrifugation was used to isolate micro bubbles with a mean diameter of approximately 3 microns [13]. Micro bubbles were coated with streptavidin (Sigma, St Louis, S4762) by incubating 1×10^9^ micro bubbles with 13 µg of streptavidin in PBS. Unbound streptavidin was removed by three sequential washes with PBS and streptavidin-coated micro bubbles were stored at a concentration >1×10^9^ micro bubbles/ml at 4°C until needed.

### Determining concentration and size-distribution of ultrasound contrast agents

The size distribution and concentration of our various ultrasound contrast agents were measured using single particle optical sizing in an Accusizer 780AD (Particle Sizing Systems, Port Richey, FL). Concentrations were reported in particles per ml and particle diameters were reported in microns.

### SFRP2-targeted UCA

Three polyclonal antibodies (two raised in goat and one in rabbit) against different epitopes of SFRP2 were purchased from Santa Cruz Biotechnology (Santa Cruz, CA): sc-7426, sc-13940 and sc-31574. These antibodies were biotinylated using EZ-Link™ Sulfo-NHS-LC-Biotinylation Kit catalogue #21435 (Thermo Scientific, Rockford, IL) according to the manufacturer's instructions by the Immunology Core Facility at the University of North Carolina at Chapel Hill. Biotinylated antibodies were incubated with streptavidin-coated microbubbles and unbound antibodies were removed by three sequential washes with PBS. SFRP2-targeted micro bubbles were stored at 4°C at a concentration >1×10^9^ micro bubbles/ml until needed.

### Non-targeted control ultrasound contrast agent

Biotinylated polyclonal antibodies raised in either rabbit or goat against chicken IgY were purchased from Bethyl Laboratories (Montgomery, TX) to serve as a control IgG mixture for the polyclonal antibodies to SFRP2. The non-targeted control ultrasound contrast agents were prepared by incubating a (2∶1) mixture of the biotinylated goat to biotinylated rabbit antibodies with streptavidin-coated contrast agent as described above.

### Verifying biotinylation of anti-SFRP2 and anti-chicken IgY by PAGE

Biotinylated SFRP2 and control antibodies were incubated with and without streptavidin prior to polyacrylamide gel electrophoresis (PAGE) through a 10% bis-tris gel (Invitrogen, Carlsbad, CA) according to manufacturer's protocol. The gel was stained with Coomassie Blue R-250 and the staining intensity and electrophoretic mobility of biotinylated antibodies bound to streptavidin was compared to that of biotinylated antibodies alone. An increase in the apparent molecular weight of the antibody in the presence of streptavidin demonstrated the antibody's ability to bind to streptavidin.

### Establishment of angiosarcoma allografts in vivo

Six week-old male nude mice were injected s.c. in their right hind limb with 1×10^6^ SVR angiosarcoma cells. Tumors reached ∼7 mm in length after one week of growth.

### Molecular imaging of SFRP2 expression with SFRP2-targeted ultrasound contrast agent

All ultrasound B-mode images were collected at 15 MHz using a 15L8 linear array transducer with a Siemens imaging system (Acuson Sequoia 512, Mountain View, CA) to provide images for selecting the region of interest (ROI) in each imaging plane and to measure tumor volume. CPS mode, a nondestructive contrast-specific imaging technique operating at 7 MHz (mechanical index = 0.18, CPS gain = −3 dB) was used to image targeted and control UCAs.

Molecular imaging of SFRP2 expression was performed with our SFRP2-targeted contrast agent as described previously [14]. Briefly, a 3-dimensional (3D) scan of the angiosarcoma tumor was performed in B-mode to record the outline of the tumor. 5×10^6^ non-targeted streptavidin-coated micro bubbles in approximately 50 µl of saline were injected into the tail vein of nude mice with angiosarcoma tumors. The perfusion of the tumor and surrounding tissue by contrast agents was captured in Cadence mode. Approximately 18 minutes were required for all free-flowing contrast agents to clear from the vasculature. At this point a 3D scan of the tumor and surrounding tissue was recorded in Cadence mode to capture signal from UCA that remained within the tumor. A baseline 3D scan was acquired after destroying contrast agents retained within the tumor with a high-energy D color scan. SFRP2-targeted micro bubbles (5×10^6^ micro bubbles in ∼50 µl of saline) were used in an identical manner to determine the expression of SFRP2 within the angiosarcoma tumors.

### Immunohistochemistry

Tumors were fixed in paraffin, sectioned, and immunohistochemistry was performed as previously described [15] using SFRP2 (1∶200 dilution) as the primary antibody. Control tumor sections were processed similarly, but without the primary antibody.

### Statistics

Results were expressed as a control-normalized, fold-increase of baseline-subtracted average volumetric pixel intensity ± SEM. A two-tailed, paired t-test was used to compare SFRP2-targeted to control-targeted imaging. A two-tailed, unpaired t-test was used to compare control-targeted imaging performed in independent experiments. To determine if there was a statistically significant relationship between tumor volume and SFRP2-targeted imaging signal the Pearson correlation coefficient (r) and its p-value were calculated. All statistical analyses were performed and all plots were created using GraphPad Prism version 5.0 d for Mac OS X, GraphPad Software, San Diego California USA, (www.graphpad.com).

## Results

### Verifying Biotinylation of polyclonal anti-SFRP2 and anti-chicken IgY

To verify successful biotinylation of SFRP2 and control antibodies, PAGE analyses were performed under non-reducing conditions because the anti-chicken IgY antibody mixtures contained a carrier protein with an apparent molecular weight of ∼50 kDa which hindered observation of the reduced heavy chain. Under non-reducing conditions the ∼150 kDa antibody was clearly present in the absence of streptavidin and shifted to a higher apparent molecular weight after incubation with streptavidin. Likewise, the apparent molecular weight of the biotinylated SFRP2 antibody was increased in the presence of streptavidin, verifying its ability to bind to streptavidin-coated contrast agent.

### Size distribution of SFRP2-targeted Ultrasound Contrast Agent

**Figure 1:**
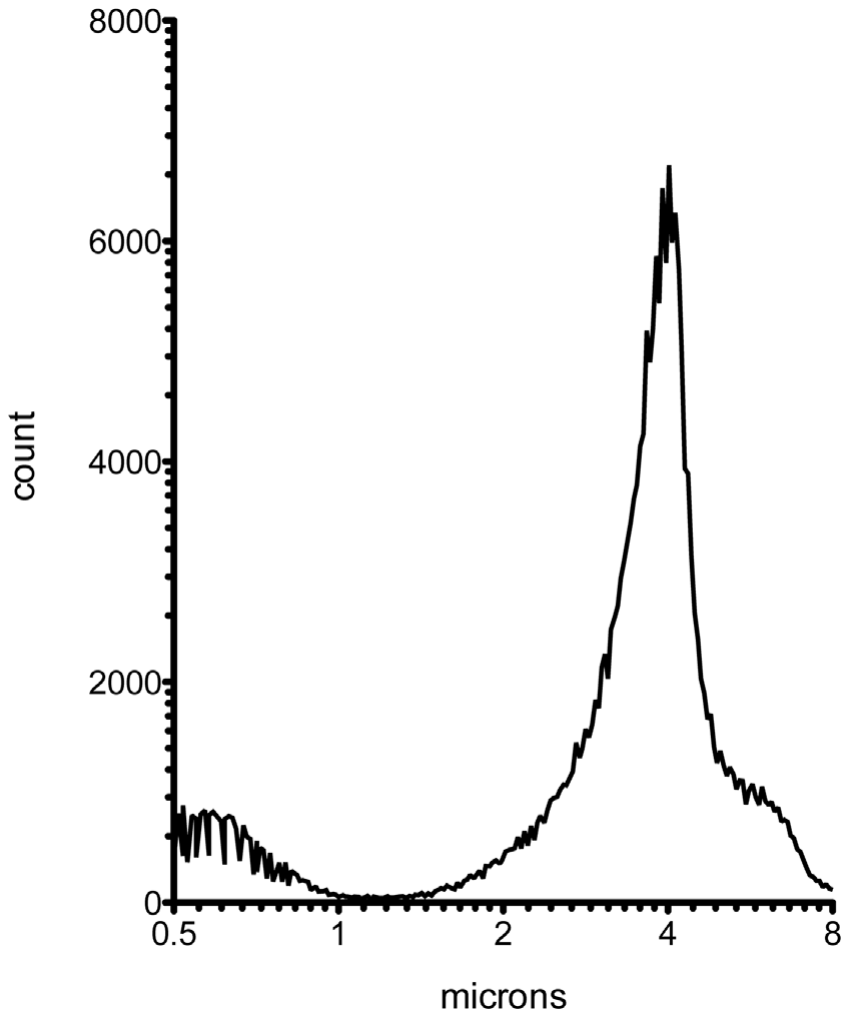
Size distribution of ultrasound microbubble contrast agent bound to SFRP2 antibody via a streptavidin bridge. Microbubble contrast agent containing biotinylated lipid was size-sorted by differential centrifugation, prior to sequential incubations with streptavidin and the SFRP2 antibody mixture. Aliquots of the contrast agent were used to determine the microbubble concentration and size distribution (Table 1).

The size distribution of microbubbles in targeted or non-targeted contrast agent did not change after addition of streptavidin and biotinylated antibodies. The average diameter of the targeted and non-targeted microbubbles was ∼3 µm with a mode of ∼4 µm and a median diameter of between ∼3–4 µm. The histogram in Fig. 1 shows the distribution of diameters in a preparation of SFRP2-targeted contrast agent and is representative of both the targeted and control contrast agent preparations. Size parameters for targeted and non-targeted contrast agents used in this study are presented in Table 1.

**Table 1:** Size-distribution of targeted and control contrast agent diameters (µm).

Contrast agent	Mean	Stdev	Mode	Median
SFRP2-targeted	3.6	1.4	4.0	3.8
anti-chicken IgY control	2.9	1.2	3.9	3.0
Streptavidin control	3.5	1.6	4.2	3.7

### SFRP2-targeted contrast agent detected SFRP2 expression in SVR tumor allografts

**Figure 2:**
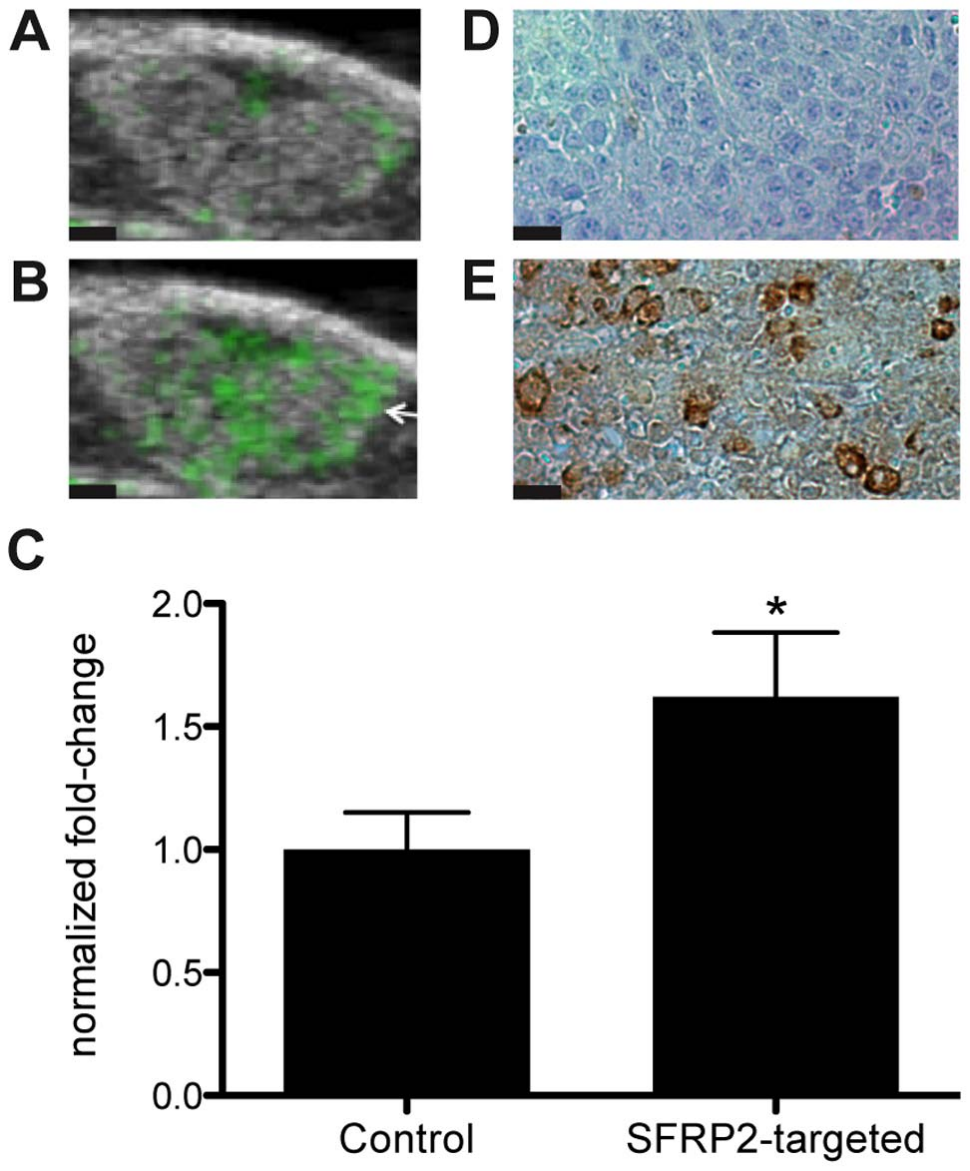
SFRP2 -targeted microbubbles bound specifically to vasculature within angiosarcoma. B-mode images of the SVR angiosarcoma tumors were overlaid in green with molecular images of (A) control streptavidin loaded microbubbles or (B) SFRP2 -targeted microbubbles after three-dimensional molecular imaging. The average pixel intensity observed for SFRP2 -targeted imaging was significantly higher (*p = 0.003, n = 13, paired t-test, two-tailed) than observed for the streptavidin control (C). Immunohistochemistry demonstrated high levels of expression for SFRP2 in angiosarcoma (D). Black scale bars in panels A and B represent 1 mm. Black scale bars in panels D and E represent 35 µm.

SFRP2 -targeted contrast agent detected tumor vasculature with significantly more signal intensity than control streptavidin-coated contrast agent (Fig. 2): the normalized fold-change was 1.6±0.27 (n = 13, p = 0.0032). After allowing all freely flowing contrast agent to be cleared from the circulation, our targeted contrast agent was retained only in the vasculature within the borders of the allograft, surrounding tissue had no significant echogenicity.

**Figure 3:**
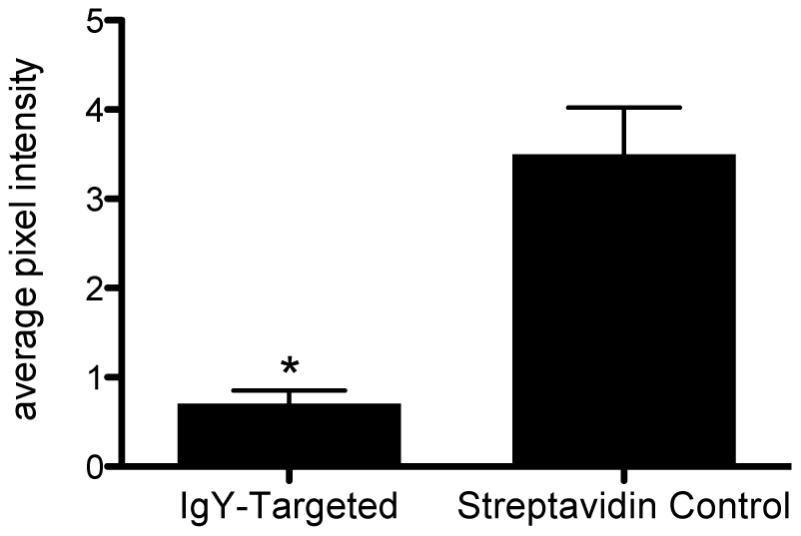
Microbubbles targeted with anti-chicken IgY were retained within angiosarcoma vasculature at significantly lower levels than microbubbles loaded with streptavidin. Streptavidin-coated microbubbles were bound to a mixture of biotinylated anti-chicken IgY (raised in rabbit and goat) to produce anti-chicken IgY control microbubbles. Three-dimensional molecular imaging with the anti-chicken IgY control microbubbles resulted in significantly lower average pixel intensity (*p = 0.0002, n = 10, unpaired t-test, two-tailed) than observed with microbubbles coated only with streptavidin.

To serve as a control IgG mixture for the polyclonal antibodies to SFRP2, in a separate experiment we compared the control streptavidin-coated contrast agent to an immunoglobulin control streptavidin-contrast agent. We evaluated the retention of control streptavidin-coated contrast agent coated with a 2∶1 mixture of biotinylated goat anti-chicken IgY and biotinylated rabbit anti-chicken IgY. We compared the baseline-subtracted average pixel intensity of control streptavidin-coated contrast agent to anti-chicken IgY-contrast agent using an unpaired, two-tailed t-test. The anti-chicken IgY-contrast agent was retained within the tumor vasculature at significantly lower levels than the streptavidin-coated contrast agent (p = 0.0002, Fig. 3). The anti-chicken IgY-contrast agent had an average pixel intensity 5-fold lower than the streptavidin-coated contrast agent. This demonstrates that immunoglobulin is not responsible for the retention of the SFRP2 contrast agent in the vasculature. Accordingly, we calculated that the SFRP2-targeted contrast agent would have average baseline-corrected pixel intensity 8-times higher than the control anti-chicken IgY-contrast agent.

### Immunohistochemistry shows SFRP2 present in SVR angiosarcoma

To demonstrate that the SVR angiosarcoma expresses SFRP2, formalin-fixed tumors were stained with a polyclonal antibody to SFRP2. This verifies that SFRP2 localizes to vessels within the angiosarcoma (Fig. 2).

### SFRP2 – targeted contrast agent is specific for tumor vasculature

**Figure 4:**
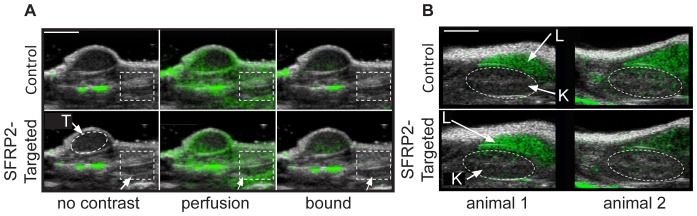
Modified microbubble contrast agents were not retained at significant levels in nonmalignant vasculature. B-mode images (black and white) are shown overlaid with CPS-mode images (green). CPS-mode is sensitive to ultrasound signal typically produced by microbubbles oscillating within an ultrasound field. (A) In the absence of ultrasound contrast agent (no contrast) there was no CPS-mode signal within the region of interest (dotted rectangle) outside of the tumor margins. Tissue artifacts generated the CPS-mode signal observed in the absence of contrast agent. Contrast agent freely flowing through both tumor and non-tumor vasculature generated CPS-mode signal throughout the field of view (panel A, perfusion, middle frames) with either streptavidin-coated (control, upper frames) or SFRP2- targeted ultrasound contrast agent (lower frames). No signal remained within the region of interest drawn outside of the tumor margins after allowing all freely flowing contrast agent to be cleared from the vasculature, while SFRP2 specific signal was retained within the tumor margins. (B) Modified microbubble contrast agents were not retained within kidney vasculature. Freely flowing streptavidin-loaded microbubbles (panel B, control, upper frames) or SFRP2 - targeted microbubbles (panel B, lower frames) were allowed to clear from the vasculature prior to three-dimensional molecular imaging. Single frames are shown from two different animals (animal 1 or animal 2). The dotted oval region of interest represents the location of the kidney (K) and there was no significant difference in average pixel intensity after injection of either streptavidin-loaded or SFRP2 -targeted microbubbles. In contrast, the liver (L) retained both modified microbubble contrast agents to a high degree. White scale bars in panels A and B represent 5 mm.

The signal from control streptavidin-coated contrast agent and SFRP2-targeted contrast was apparent throughout the tumor and surrounding normal tissue while these reagents were freely circulating through the vasculature. After allowing all freely flowing contrast agent to be removed from circulation, video signal was significantly lower in the normal tissue surrounding the tumor than within the tumor (Fig. 4a). This demonstrated that the SFRP2-targeted contrast agent and the control contrast agent did not bind significantly within normal vasculature. Therefore, the SFRP2-targeted contrast agent is specific for tumor vessels compared to normal vessels.

In addition, we examined the video intensity in the kidney and in the liver (Fig. 4b). We found that both the SFRP2-targed contrast agent and the control contrast agent were retained within the liver, resulting in intense echogenicity. On the other hand, kidney was largely devoid of echogenicity with no significant difference between the control contrast agent and the SFRP2-contrast agent, again demonstrating specificity for tumor vessels.

### SFRP2 -targeted contrast agent intensity increases with tumor volume

**Figure 5:**
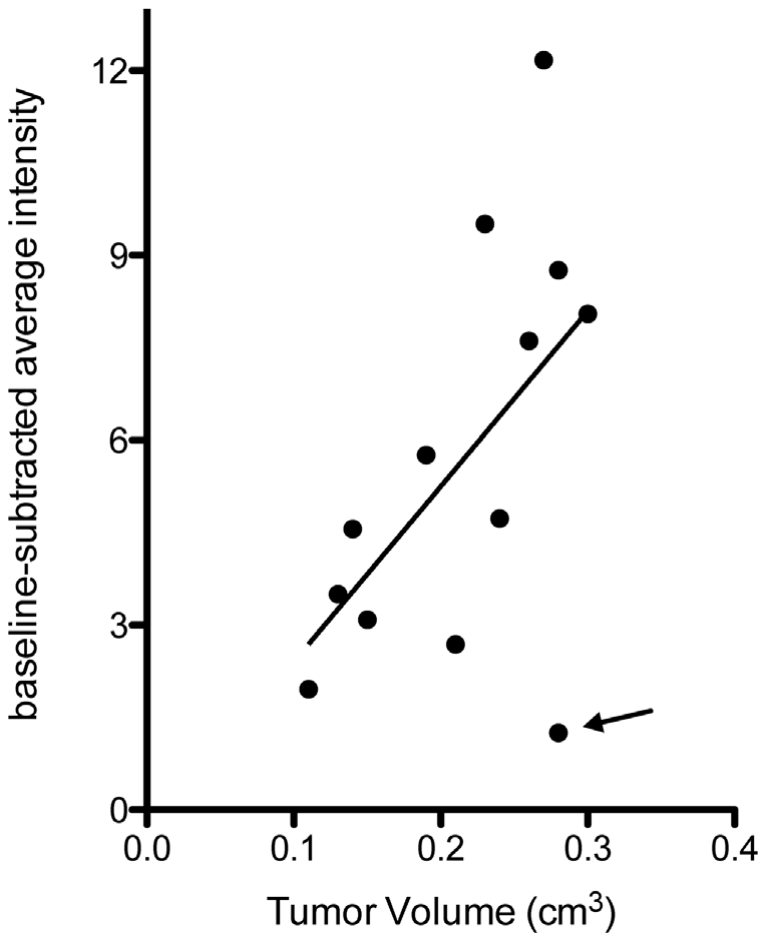
Video intensity from SFRP2 -targeted microbubble contrast agent correlated significantly with SVR angiosarcoma tumor volume. The baseline-subtracted average pixel intensity for each tumor was plotted against tumor volumes determined using our three-dimensional B-mode scans. Only 1 of 13 animals was observed to have higher signal for the streptavidin control than for the SFRP2 -targeted microbubbles (indicated by arrow). Correlation analysis showed a significant positive relationship between SFRP2 video signal and tumor volume as both increased together whether analyzed with (p = 0.048, Pearson r = 0.56) or without (p = 0.003, Pearson r = 0.78) the single “outlier” animal. Linear regression was used to determine the best-fit line through all data points to represent this relationship.

When we plotted average pixel intensity obtained from SFRP2-targeted contrast agent against tumor volume (Fig. 5), we found that in general, average pixel intensity increased as tumor volume increased (n = 13). Only one of thirteen animals examined had higher average pixel intensity for the control-contrast agent than for the SFRP2 -targeted contrast agent (indicated by the arrow in Fig. 5). Correlation analysis showed a highly significant relationship (p = 0.003, Pearson r = 0.78) between tumor volume and SFRP2-targeted video signal when omitting the aforementioned “outlier.” Even when this data point was included in the correlation analysis, there was a significant relationship between tumor volume and SFRP2- targeted video signal (p = 0.048, Pearson r = 0.56) as illustrated by the best-fit line in Fig. 5. In the range of tumor volumes investigated, as tumors increased in volume SFRP2 expression increased.

## Discussion

SFRP2 is a tumor endothelial marker with increased expression in tumor vessels compared to normal vessels [2], and is expressed in a wide variety of human tumors including angiosarcoma. We created an SFRP2 targeted ultrasound contrast agent that is retained in tumor vasculature but does not bind to normal vessels. This novel contrast agent could be useful to help differentiate between benign and malignant lesions on ultrasound, increasing the specificity of ultrasound for cancer detection.

The molecular imaging of SFRP2 provides a tool to understand the role of this secreted factor during tumor progression. There has been controversy in the literature as to whether SFRP2 is a tumor suppressor or promoter of tumor growth. SFRP2 has been implicated in binding to Wnts, thereby blocking Wnt binding to Frizzled receptors, and resulting in inhibition of β-catenin activation [16]. This, in combination with data showing that SFRP2 is hypermethylated in certain tumors [17]–[19]has led to an assumption that SFRP2 is a tumor suppressor. However, there is a discrepancy in which several studies have shown that SFRP2 is an agonist (rather than an antagonist) of β-catenin [20]–[24] suggesting the reverse: that SFRP2 may promote tumor growth. Further evidence to support this theory is that SFRP2 has been found to be produced by the majority of malignant glioma cell lines, and SFRP2 overexpressing intracranial glioma xenografts were significantly larger than xenografts consisting of control cells in nude mice [25]. Transient transfection of SFRP2 in renal cell carcinoma has been shown to increase tumor growth *in vivo* [26]. We recently reported the development of a murine monoclonal antibody to SFFRP2 that inhibits the growth of both SVR angiosarcoma and triple negative breast cancer in vivo.

Through our assumption that the anti-SFRP2 molecularly targeted contrast agents bind to endothelium in proportion to the amount of SFRP2 expressed, we hypothesize that we can detect whether SFRP2 expression increases or decreases during tumor growth. An increase in SFRP2 as tumor volumes increase would support the theory that SFRP2 stimulates tumor growth; and a decrease in SFRP2 while tumors grow would support the theory that SFRP2 is a tumor suppressor. Our data show that SFRP2 expression increased as tumor volume increased in vivo, providing further support for the role of SFRP2 as a stimulator of tumor growth.

Challenges to anti-tumor and anti-angiogenic compound development and clinical implementation [27] include lack of knowledge of early response to therapy and resistance that can develop to therapy [28]. Molecular imaging of SFRP2 expression may provide a rapid, non-invasive method to monitor tumor regression during therapy for angiosarcoma, and contributes to our understanding of the biology of SFRP2 during tumor progression.
